# The Kousso—A New Anthelmintic

**Published:** 1851-03

**Authors:** Jonathan Pereira


*The Kousso—a New Anthelmintic.* By Jonathan Pereira, M. D., F. R. S.—The Kousso, or Brayera Anthelmintica, is an Abyssinian tree twenty feet high. Branches round, rusty, tomentose-villose, marked by the annular cicatrices of the fallen leaves. Leaves crowded, alternate, interruptedly imparipinnate and sheathing at the base. Leaflets oblong, or elliptical lanceolate, acute, serrate, villose at the margin and on the nerves of the under surface. Stipules adnate to the petoile, which is dilated at the base and amplexicaul. Flowers diœcious, small, greenish, and becoming purple; repeatedly dichotomous; the pedicels with an ovate bract at the base.

The so-called male flowers may be regarded as hermaphrodite flowers, inasmuch as the carpels are well developed. The female flowers are somewhat different in their structure. The outer segments of the calyx are much more developed than in the female flowers, and are four or five times larger than those of the inner row, and are placed somewhat below them; the petals are entirely wanting; the stamina are rudimentary and sterile. The ripe fruits are unknown.

The tree grows in Tigre, Agame, and Shoa; it is cultivated everywhere, and Dr. Beke writes that the tree is "found throughout the entire table-land of North-Eastern

Abyssinia, but appears to require an elevation of upwards of six thousand (perhaps of seven thousand) feet for its growth. Where I found it most luxuriant was in the vicinity of the source of the river Abai (Bruce's Nile,) at an elevation of close upon nine thousand feet. Tigre, the northern portion of Abyssinia, being, on the whole, of lower elevation than the rest of that country, the tree is only found there in a few places."

Bruce describes the flowers as being of a greenish color, tinged with purple, and when fully blown, of a deep red or purple. The petals, he says, are white.

*Preparation.*—Mr. Johnston states that the kousso is gathered for medicinal purposes before the seeds are quite ripe, whilst still a number of florets remain unchanged. The bunches are suspended in the sun to dry, and if not required for immediate use are deposited in a jar.

*Pharmacography.*—The author has seen only one package of kousso; this was kindly opened in his presence by M. Simond, of the firm of Caylits, Simond & Co., the agents of M. Rochet d'Hericourt. It was a deal box containing about 30 lbs. of the dried flowers, wrapped up in a large skin of red leather. On removing the lid of the box and untying the leather package, the fragrant or balsamic odor of the dried flowers was very powerful. It appeared to him to be somewhat similar to the combined odors of tea, hops, and senna leaves. The flowers had apparently undergone no preparation beyond that of desiccation. The bunches of flowers were perfect and unbroken, though of course compressed. The general color of the dried mass was greenish-yellow; but when the flowers were more closely examined, the edges of the petals were seen to have a reddish or purplish color. The taste of the dried flowers is at first not very marked, but after a few minutes a feeble, senna-like, acrid, unpleasant taste becomes perceptible. By soaking the dried flowers in water, they may be unfolded sufficiently to determine their botanical characters which have been already described. When submitted to microscopic examination, the hairs are perceived to be simple lymphatic hairs, tapering at the distal extremity. In Abyssinia, two sorts of kousso are distinguished, viz., first, the *red* kousso produced by the female flowers; secondly, the male flowers known as *kousso esels.* In commerce the two sorts are always mixed together.

*Adulteration.*—Considering the enormous price (about 1*l.* 15*s.* per ounce) at which kousso has hitherto been sold in Paris, and the very limited quantity originally supplied by M. Rochet d'Hericourt, it cannot be surprising that the article should be extensively adulterated. Indeed, the author has been assured, on credible authority, that the powder now selling as kousso is, in fact, the powder of pomegranate bark, and that legal proceedings have been commenced in Paris to put a stop to the fraud, which is well calculated to injure the reputation of the genuine Abyssinian remedy. He has no doubt but that the microscope would readily detect the substitution; but the surest way of obtaining the genuine article is, to purchase the dried flowers in the entire state, not in the form of powder.

Although it is not improbable that the anthelmintic property of kousso may in part depend on tannin (since the pomegranate bark, which contains this principle in abundance, is, like kousso, also an anthelmintic,) yet what may be termed the peculiar property of the kousso probably resides chiefly in the bitter acrid resin. This is soluble in alcohol and in ether, and appears to be a neutral body, manifesting neither distinct alkaline nor acid properties.

By boiling the dried plant in water a fragrant odor is evolved. No doubt this, as well as the odor of the dried plant itself, depends on the presence of a *volatile oil*, of which, however, no mention is made in Wittstein's analysis, the oil being present in too small a quantity to admit of its collection when small quantities of the flowers are operated on. It is not improbable that the anthelmintic properties may, in part, depend on this oil; for Schimper states, that in Abyssinia the plant is considered to have lost its anthelmintic powers in the third year after its collection. In Europe, however, it retains its powers for a longer period (on account of the cooler climate?) for the flowers which have been used for all the recent experiments have been collected more than four years, and we are told in the shop-bill of a Parisian pharmacien, that they may be kept for an indefinite period.

An infusion or a decoction of kousso strikes a dark green olive tint with a solution of the sesqui-chloride of iron.

*Medicinal Properties.*—Neither botanical characters, sensible qualities, nor chemical composition, would have induced us to suspect that kousso possesses the valuable anthelmintic properties which experience has shown that it does. The general and prevailing quality of the rosaceæ is astringency, dependent on the presence of tannic and gallic acids. This is observed in the flowers (*e.g.* rose-petals) as well as in other parts of the plants. In this quality kousso agrees with its congeners. But it can scarcely be on this that its vermifuge property solely depends; otherwise the rose-petals, or other equally powerful astringent, would be as effective in expelling worms as the Abyssinian flowers. But in rosaceæ, as in many other families of the vegetable kingdom, anomalies exist, and to this head we must, for the present, be content to refer kousso.

Our confidence in the anthelmintic properties of kousso rests, then, on experience only: and the evidence on this point is very strong. All modern travellers in Abyssinia are agreed on the great success of the remedy on the natives of that country; and the experience of physicians in France, England, Germany, and Switzerland, confirms the favorable reports made by those who have seen the kousso used in its native country.

In Paris it has been employed with great success by Chomel and Sandras, as well as by numerous other distinguished physicians. In London, our experience of it is much more limited; but the successful results of its use in King's College Hospital, in the hands of Drs. Budd and Todd, and of Dr. Gull, in Guy's Hospital, confirm the favorable reports of its efficacy which had reached this country from abroad.

The physiological effects of kousso are not in general very great. Sometimes it excites a slight sensation of heat, nausea, or even vomiting, creates thirst, and frequently, perhaps usually, a gentle action on the bowels. But the latter is commonly so slight, that in a considerable number of cases it is necessary to follow its administration by a mild purgative. It is obvious, therefore, that the efficacy of kousso as an anthelmintic does not depend on its purgative or evacuant influence, but on its poisonous or toxic action on the worm; in fact, it is a true *vermicide.* In one case, that of a woman in France, it brought away ten worms, of which one only manifested evidences of vitality, and that for a few minutes only.

Kousso appears to be an effective anthelmintic in both kinds of tape-worm; viz., the *tænia solium* and *bothriocephalus latus.* In most of the reported successful cases, the *tænia solium* was the parasite expelled; but in one of Chomel's cases, the worm which was evacuated was the *bothriocephalus latus*, and the author is informed, that kousso has proved most effectual in Switzerland, where, as is well known, the *bothriocephalus* is the prevailing tape-worm.

The dealers in kousso assert that one dose will, in every case, effect the radical cure of the tape-worm. But this must be obviously an error. Even supposing that it invariably destroys all the worms in the alimentary canal at the time of its exhibition, it can in no way prevent their recurrence, provided the patient retains his predisposition, which there is no reason to suppose is affected by the kousso, and is subjected to the same influence. It certainly does not radically cure the Abyssinians: since, as several writers tell us, they resort to this remedy monthly. Schimper, the governor of Adoa, says it does not completely expel the tænia, or at least rarely does so. But he adds, that possibly in Europeans, in whom the verminous disposition is not so pronounced as in the Abyssynians, it may perhaps act in a more complete manner. In the Abyssinians this verminous disposition is innate, and is dependent, he adds, on the regimen which they adopt.

Hitherto the great drawback to the use of kousso has been the difficulty of procuring the remedy, and its enormous cost. At the time when it could be purchased in Paris, its price was 1*l.* 15*s.* per ounce, or 17*s.* 6*d.* per dose. M. Rochet d'Hericourt, the sole holder of the medicine at the present time, refuses to sell any quantity less than his entire stock, at the rate of one guinea per ounce. His nephew tells me that his uncle possesses 1400 lbs of it, which, at one guinea per ounce, will cost 22,400 guineas. The impossibility of effecting a sale on such terms will, the author doubts not, ultimately compel the holder to reduce his demands to something approaching to reason. It does not appear that the remedy is very costly in Abyssinia. Schimper, in writing from Adoa, in Abyssinia, says that it is found in commerce at a very low price. At Yangaro (commonly called Zingaro) the sovereign has the exclusive use of it, his subjects being prohibited from employing it; but in other parts free trade in kousso is permitted. Considering the frequency and rapidity of our communications with Egypt, to which place, according to Dr. Brayer, kousso is conveyed by caravans, no difficulty, the author apprehends, will be experienced in obtaining an abundant supply of it. Its present price is a virtual prohibition of its use.

The flavor, though not very strong, is by no means agreeable; and is sufficiently powerful in some patients to create disgust and excite vomiting. In one case, under M. Chomel, the whole of the remedy was rejected by vomiting. No ill effects have resulted from its use in this country, nor has the author met with any statement of its injurious action, except in Mr. Johnson's "Travels in Southern Abyssinia," where it is stated that its "operation is speedy and effectual; and to judge by the prostration of strength it occasioned in my servants when they employed this medicine, it must be dreadfully severe. I can answer for this, that it occasions frequent miscarriages, often fatal to the mother, and even men have been known, after a large dose, to have died the same day from its consequences. I am, therefore, surprised at the noise which this remedy has occasioned the last few years in Europe, as if it promised to be a valuable addition to our materia medica. This, I conceive, can never be; for no civilized stomach could bear the bulk of the drug necessary to produce its effects. Even in Abyssinia it is but barely tolerated; and let another remedy equally efficacious for dislodging tape-worm be introduced into that country, and the use of kousso will be soon abandoned. In fact, several other vegetable productions are now employed to escape the punishment of a dose of this violent cathartic."

*Administration.*—Both Bruce and Schimper tell us that the Abyssinians take a handful of the dried flowers as a dose. In Paris the dose has varied from four to six drachms. In general, however, half an ounce (troy weight) is considered a dose for an adult. For different ages the doses are thus adjusted:

| | | |
|---|---|---|
| Adults ........................ | 1 dose | =240 grs. ($\frac{1}{2}$ oz.) |
| Children from 7 to 12 years.. | $\frac{2}{3}$ of a dose | =160 " |
| " " 3 to 7 " ... | $\frac{1}{2}$ " | =120 " |
| " not exceeding 3 " ... | $\frac{1}{3}$ " | = 80 " |

The kousso should be taken in the morning fasting. The only preparation necessary is, that the last meal of the preceding evening should be slight. The evacuation of the bowels by a mild purgative or a lavement is also desirable. The mode of administering the remedy is as follows:—the powdered flowers are to be mixed with lukewarm water, for an adult about ten ounces, and allowed to infuse for a quarter of an hour. A little lemon juice is then to be swallowed, and the infusion being stirred up, the whole is taken, liquid and powder, at two or three draughts, at short intervals, being washed down by cold water and lemon-juice. To promote the operation, tea, without sugar or milk, may be taken. In three or four hours, if the remedy has not operated, a dose of castor oil, or a saline purgative, should be administered.—*Dublin Med. Press and Pharmaceutical Journal*, from *Ranking's Abstract.*

